# Multiplex Genetic Engineering Exploiting Pyrimidine Salvage Pathway-Based Endogenous Counterselectable Markers

**DOI:** 10.1128/mBio.00230-20

**Published:** 2020-04-07

**Authors:** Lukas Birštonas, Alex Dallemulle, Manuel S. López-Berges, Ilse D. Jacobsen, Martin Offterdinger, Beate Abt, Maria Straßburger, Ingo Bauer, Oliver Schmidt, Bettina Sarg, Herbert Lindner, Hubertus Haas, Fabio Gsaller

**Affiliations:** a Institute of Molecular Biology/Biocenter, Innsbruck Medical University, Innsbruck, Austria; b Research Group Microbial Immunology, Leibniz Institute for Natural Product Research and Infection Biology-Hans Knöll Institute, Jena, Germany; c Institute of Neurobiochemistry/Biocenter, Innsbruck Medical University, Innsbruck, Austria; d Transfer Group of Antiinfectives, Leibniz Institute for Natural Product Research and Infection Biology-Hans Knöll Institute, Jena, Germany; e Institute of Cell Biology/Biocenter, Innsbruck Medical University, Innsbruck, Austria; f Institute of Clinical Biochemistry/Biocenter, Innsbruck Medical University, Innsbruck, Austria; Universidade de São Paulo

**Keywords:** endogenous selectable markers, genetic engineering, pyrimidine salvage pathway, targeted genomic integrations

## Abstract

Selectable markers are indispensable for genetic engineering, yet their number and variety are limited. Most selection procedures for prototrophic cells rely on the introduction of antibiotic resistance genes. New minimally invasive tools are needed to facilitate sophisticated genetic manipulations. Here, we characterized three endogenous genes in the human fungal pathogen Aspergillus fumigatus for their potential as markers for targeted genomic insertions of DNAs of interest (DOIs). Since these genes are involved in uptake and metabolization of pyrimidines, resistance to the toxic effects of prodrugs 5-fluorocytosine and 5-fluorouracil can be used to select successfully integrated DOIs. We show that DOI integration, resulting in the inactivation of these genes, caused no adverse effects with respect to nutrient requirements, stress resistance, or virulence. Beside the individual use of markers for site-directed integration of reporter cassettes, including the 17-kb penicillin biosynthetic cluster, we demonstrate their sequential use by inserting three genes encoding fluorescent proteins into a single strain for simultaneous multicolor localization microscopy. In addition to A. fumigatus, we validated the applicability of this novel toolbox in Penicillium chrysogenum and Fusarium oxysporum. Enabling multiple targeted insertions of DOIs without the necessity for exogenous markers, this technology has the potential to significantly advance genetic engineering.

## Abstract (precis)

This work reports the discovery of a novel genetic toolbox comprising multiple, endogenous selectable markers for targeted genomic insertions of DNAs of interest (DOIs). Marker genes encode proteins involved in 5-fluorocytosine uptake and pyrimidine salvage activities mediating 5-fluorocytosine deamination as well as 5-fluorouracil phosphoribosylation. The requirement for their genomic replacement by DOIs to confer 5-fluorocytosine or 5-fluorouracil resistance for transformation selection enforces site-specific integrations. Due to the fact that the described markers are endogenously encoded, there is no necessity for the exogenous introduction of commonly employed markers such as auxotrophy-complementing genes or antibiotic resistance cassettes. Importantly, inactivation of the described marker genes had no adverse effects on nutrient requirements, growth, or virulence of the human pathogen Aspergillus fumigatus. Given the limited number and distinct types of selectable markers available for the genetic manipulation of prototrophic strains such as wild-type strains, we anticipate that the proposed methodology will significantly advance genetic as well as metabolic engineering of fungal species.

## INTRODUCTION

**FIG 1:**
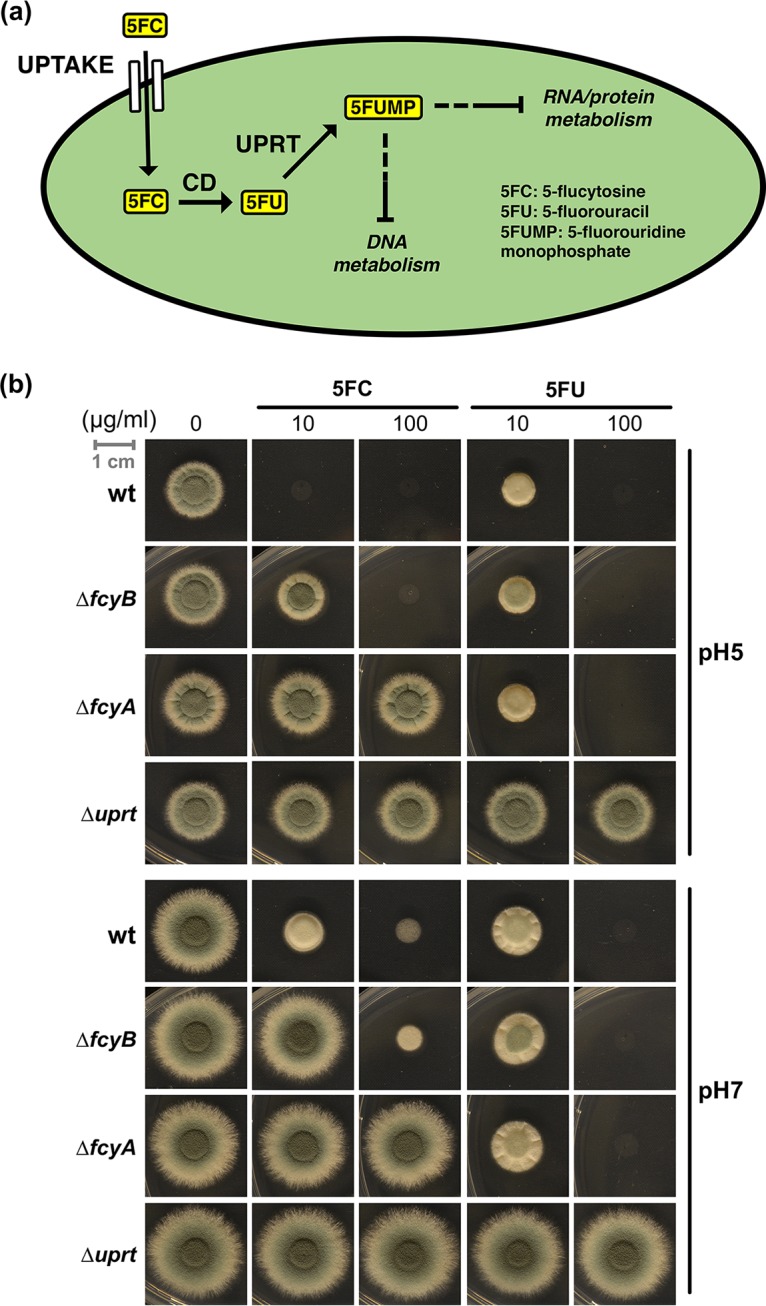
Metabolization of 5FC and associated genetic factors in A. fumigatus. (a) After uptake, 5FC is converted to 5FU by the enzyme cytosine deaminase (CD). Subsequently, 5FU is phosphoribosylated to 5FUMP by uracil phosphoribosyltransferase (UPRT). 5FUMP is further metabolized into RNA or DNA nucleotides that interfere with DNA, RNA, as well as protein metabolism (12). (b) Inactivation of genes encoding components involved in uptake (Δ*fcyB*), CD (Δ*fcyA*), and UPRT (Δ*uprt*) activity in A. fumigatus leads to different degrees of 5FC and 5FU resistance. For plate growth-based susceptibility testing, strains were point inoculated on solid AMM containing different levels of 5FC and 5FU. Images were acquired after 48 h of incubation at 37°C. Dashed lines indicate several enzymatic steps.

Genetic engineering commonly involves the introduction of DNA of interest (DOI) into a target cell, followed by its integration into the genome. However, the efficiency of such genetic transformations is typically very limited. Therefore, selection of successfully modified cells usually involves cotransformation of selectable marker genes together with the DOI to allow growth under selective conditions. Widely used marker cassettes either compensate for the inability to synthesize vital metabolites (auxotrophic selection markers) or confer resistance to growth inhibitory compounds such as antibiotics (dominant selectable markers) (1). While auxotrophic markers are restricted to auxotrophic recipients, dominant selectable markers can be used for virtually any prototrophic recipient cell that is susceptible to the antibiotic used for selection. Hence, dominant selectable markers play a crucial role in the genetic manipulation of most wild-type cells. Examples of selectable markers commonly employed for the genetic engineering of fungi include genes conferring resistance to hygromycin B, phleomycin, pyrithiamine, kanamycin, and nourseothricin (2–4). Their expression allows growth in the presence of the corresponding antibiotic, which classifies them as positive selectable markers. Negative selectable markers, in contrast, inhibit growth of the target cells during selective conditions. In addition to the herpes simplex virus 1 thymidine kinase gene (5, 6), genes encoding cytosine deaminase (CD) and uracil phosphoribosyltransferase (UPRT) have been employed as negative selectable markers in diverse organisms (7–11). The presence of functional gene copies providing CD and UPRT activity renders host cells susceptible to prodrugs 5-fluorocytosine (5FC) and 5-fluorouracil (5FU). CD and UPRT, both enzymes of the pyrimidine salvage pathway, convert 5FC and 5FU into 5-fluorouridine monophosphate (5FUMP) (Fig. 1a). Further metabolization of 5FUMP into toxic ribo- and deoxyribonucleotides blocks cellular growth (12).

Here, we characterized the pyrimidine salvage pathway in the human fungal pathogen Aspergillus fumigatus and present its application for fungal genetic engineering. In the described technology, endogenous genes encoding 5FC uptake, CD, and UPRT serve as counterselectable markers for targeted, genomic introduction of multiple DOIs. Homologous recombination-driven replacement of marker genes by DOIs results in their inactivation, which can be selected via 5FC/5FU resistance. In addition to the individual use (e.g., integration of reporter cassettes as well as the 17-kb penicillin biosynthetic cluster), the potential sequential use of the three loci is demonstrated by the insertion of three different fluorescent-protein-encoding genes for multicolor imaging of three cellular compartments. Demonstrating its versatile applicability, the described technology was implemented in the industrial work-horse Penicillium chrysogenum and the plant pathogen Fusarium oxysporum.

## RESULTS

### Cytosine deaminase FcyA and uracil phosphoribosyltransferase Uprt are crucial for the metabolic activation of 5FC in Aspergillus fumigatus.

Metabolization of 5FC has been well-studied in the model yeast Saccharomyces cerevisiae: 5FC is converted by the CD Fcy1p to 5FU (13, 14) and subsequently phosphoribosylated to 5FUMP by the UPRT Fur1p (15). Inactivation of each of these steps resulted in 5FC resistance, whereby inactivation of Fur1p also conferred 5FU resistance (15). Orthologous proteins from S. cerevisiae (Fcy2p), A. fumigatus, and Aspergillus nidulans (FcyB) have been identified as major cellular 5FC importers (16–18).

Among other fungal species, A. fumigatus is susceptible to 5FC (19, 20) and is therefore anticipated to harbor genes encoding CD and UPRT activities in addition to 5FC uptake. BLASTP-based *in silico* predictions revealed A. fumigatus FcyA (AFUB_005410) and Uprt (AFUB_053020) as putative orthologs of *S. cerevisiae* Fcy1p and Fur1p, respectively. To analyze their role in 5FC as well as 5FU metabolism, we inactivated *fcyA* and *uprt* in the A. fumigatus strain A1160P+ (21), termed wild type (wt) here, using hygromycin and phleomycin resistance-based deletion cassettes. Due to the interdependency of 5FC activity and environmental pH (16, 19), we investigated the contribution of both enzymes, as well as FcyB, to 5FC and 5FU activity at both pH 5 and pH 7.

Plate growth-based susceptibility testing revealed that, similar to previous work (16), 5FC levels ≥ 10 μg/ml blocked wt growth at pH 5, while 100 μg/ml 5FC was required at pH 7 (Fig. 1b). Although FcyB is the major 5FC uptake protein, at 100 μg/ml 5FC, the Δ*fcyB* mutant strain was not able to grow at pH 5 and showed severe growth inhibition at pH 7. In contrast to the Δ*fcyB* mutant, the Δ*fcyA* and Δ*uprt* mutants displayed full resistance to 5FC up to 100 μg/ml, regardless of the pH. Furthermore, 100 μg/ml 5FU blocked growth of wt, Δ*fcyB*, and Δ*fcyA* strains at pH 5 as well as pH 7, while the Δ*uprt* mutant was resistant.

Our data confirm the role of FcyB as a major 5FC cellular importer but indicate the presence of additional uptake mechanisms. Similar to the orthologous proteins in S. cerevisiae, our findings reveal the essential roles of FcyA and Uprt for 5FC activity and of Uprt for metabolic activation of 5FU in A. fumigatus.

### The *fcyB*, *fcyA*, and *uprt* loci can be used for 5FC/5FU-based transformation selection.

Lack of FcyB, FcyA (CD activity), or Uprt (UPRT activity) confers resistance to 5FC (Δ*fcyB*, Δ*fcyA*, and Δ*uprt* mutants) or 5FU (Δ*uprt* mutant) (Fig. 1b), which suggested the utilization of the genes encoding these proteins as counterselectable markers for positive selection of cells with targeted integration of DOIs. Moreover, the different degrees in 5FC resistance observed for Δ*fcyB* and Δ*fcyA* indicated that 5FC can be used for selection of loss of FcyB at low 5FC concentrations (10 μg/ml) and loss of FcyA at high 5FC levels (100 μg/ml) (Fig. 1b). Selection for loss of Uprt was conducted with 100 μg/ml 5FU.

**FIG 2:**
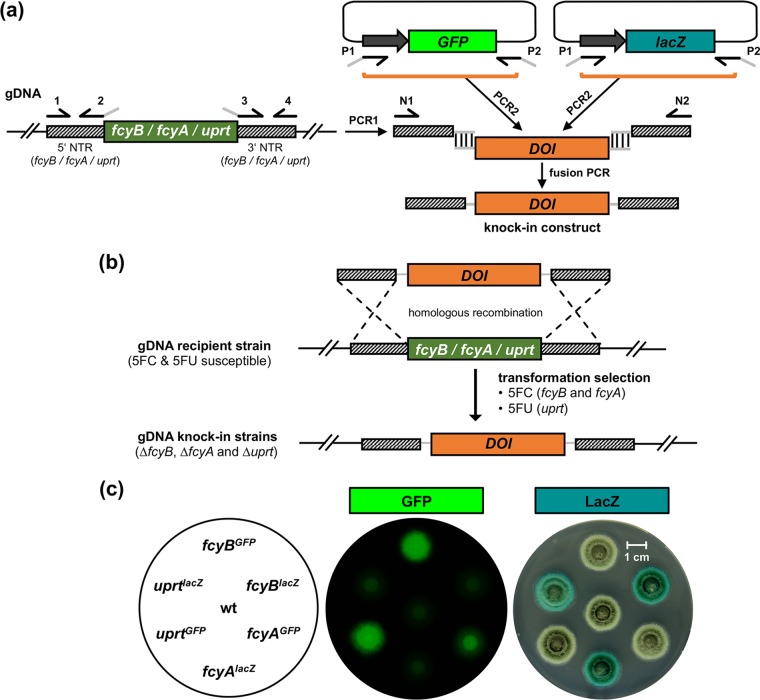
Replacement of endogenous counterselectable markers *fcyB*, *fcyA*, and *uprt* by DNAs of interest (DOIs). (a) Scheme of the generation of knock-in constructs. 5′ and 3′ nontranslated regions (NTRs) (PCR1) of the respective loci as well as the DOIs (PCR2; *GFP* or *lacZ* reporter cassette) are amplified from genomic DNA (gDNA) and plasmid DNA, respectively. Both NTRs and DOIs contain overlapping DNA (gray line) for subsequent connection via fusion PCR, yielding the knock-in constructs. (b) Double crossover homologous recombination-based replacement of *fcyB*, *fcyA*, or *uprt* by DOIs. Transformation selection was conducted using 5FC (*fcyB* and *fcyA* locus) or 5FU (*uprt* locus). (c) Visualization of GFP as well as LacZ expression in the corresponding knock-in strains after incubation on solid AMM for 48 h at 37°C.

For proof of principle, both green fluorescent protein (GFP, the sGFP[S65T] variant, here abbreviated as sGFP) (37) and β-galactosidase (LacZ) expression cassettes were used to replace *fcyB*, *fcyA*, as well as *uprt*. To achieve homologous recombination-mediated replacement of these loci with the reporter cassettes, approximately 1-kb 5′ and 3′ nontranslated regions (NTRs) of the respective gene were linked to each cassette via fusion PCR (Fig. 2a). The resulting knock-in constructs were transformed into wt protoplasts, which underwent selection for resistance to 5FC and 5FU (see above; Fig. 2b). Southern blot analyses confirmed site-specific integration of the DOIs into each of the three loci (see Fig. S1 in the supplemental material). In agreement, all knock-in strains displayed resistance phenotypes according to the respective mutation in the pyrimidine salvage pathway (compare Fig. 1b and Fig. S2). Fluorescence imaging and β-galactosidase staining confirmed the functionality of the knock-in cassettes (Fig. 2c). To analyze the transformation efficiency using individual selectable marker genes, we employed the corresponding LacZ knock-in constructs for each locus. For *fcyB*, *fcyA*, and *uprt*, 10, 27, and 13 transformants, respectively, were recovered on the corresponding selective media (Fig. S3). Out of these 10 (100%), 26 (97%) and 12 (92%) displayed LacZ activity. Southern blot analysis of 10 LacZ-positive transformants for each locus confirmed correct integrations.

10.1128/mBio.00230-20.1 FIG S1 Southern blot analysis of strains generated in this work. In each blot, a representative transformant is compared to the respective recipient strain; wt, A. fumigatus A1160P+; Pc, Penicillium chrysogenum, Fo, Fusarium oxysporum. Download FIG S1, DOCX file, 0.5 MB. Copyright © 2020 Birštonas et al. 2020 Birštonas et al. This content is distributed under the terms of the Creative Commons Attribution 4.0 International license.

10.1128/mBio.00230-20.2 FIG S2 Plate growth-based 5FC/5FU susceptibility testing of A. fumigatus GFP and LacZ knock-in strains as well as *RFP^PER^ GFP^MIT^BFP^CYT^* and its progenitor strains. Strain were point inoculated on solid AMM at both pH 5 and pH 7 and incubated for 48 h at 37°C. Resistance phenotypes of all mutants analyzed were in accordance with the absence of individual salvage activities. Download FIG S2, DOCX file, 2.6 MB. Copyright © 2020 Birštonas et al. 2020 Birštonas et al. This content is distributed under the terms of the Creative Commons Attribution 4.0 International license.

10.1128/mBio.00230-20.3 FIG S3 β-Galactosidase staining to screen for LacZ-positive transformants. After determining LacZ activities of each transformant (a), 10 transformants per locus showing LacZ-positive phenotypes (yellow numbers) were subjected to Southern blot analysis (b). Strains were grown for 48 h at 37°C on solid AMM before pouring an additional 5-ml layer of X-Gal-containing agar over the colonies. Download FIG S3, DOCX file, 2.4 MB. Copyright © 2020 Birštonas et al. 2020 Birštonas et al. This content is distributed under the terms of the Creative Commons Attribution 4.0 International license.

Taken together, 5FC- and 5FU-mediated selection allowed replacement of each of the three loci by either a *GFP* or *lacZ* expression cassette, which demonstrates the suitability of *fcyB*, *fcyA*, and *uprt* as selectable markers for targeted, integrative transformation in A. fumigatus.

### *fcyB*, *fcyA*, and *uprt* can be consecutively used for multiple genomic integrations.

Due to the fact that inactivation of *fcyB* and *fcyA* led to different levels of resistance to 5FC and inactivation of *uprt* caused resistance to 5FU (Fig. 1b), we investigated whether these marker genes can be sequentially employed for transformation selection in a single strain. As an exemplary application, we aimed to generate a strain expressing three fluorescent proteins for multicolor imaging: GFP, red fluorescent protein (RFP, mKate2) and blue fluorescent protein (BFP, mTagBFP2).

The strategy pursued and order of markers used for selection was based on the following results (Fig. 1b). (i) In contrast to the wt, the Δ*fcyB* mutant can grow in the presence of 10 μg/ml 5FC at pH 5. (ii) In contrast to Δ*fcyB*, Δ*fcyA* can grow at 100 μg/ml 5FC, which allows discrimination of Δ*fcyB* Δ*fcyA* from Δ*fcyB*. (iii) Δ*fcyB* and Δ*fcyA* are still able to import and metabolize 5FU, which is expected to allow discrimination of Δ*fcyB* Δ*fcyA* and Δ*fcyB* Δ*fcyA* Δ*uprt* in the presence of 100 μg/ml 5FU.

**FIG 3:**
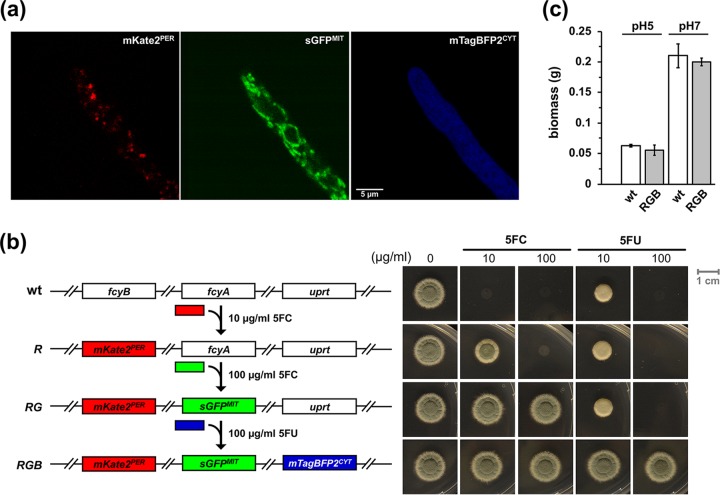
Multicolor imaging and phenotypic analysis of *RFP^PER^ GFP^MIT^ BFP^CYT^* (*RGB*) expressing three fluorescent proteins with distinct subcellular localization. (a) Using laser scanning confocal microscopy, the expression of peroxisomal RFP, mitochondrial GFP, and cytoplasmic BFP was monitored in *RGB* after incubation for 20 h in liquid AMM at 30°C. (b) Scheme illustrating the sequential integration of fluorescent proteins (left) and 5FC/5FU resistance profiles (right) of *RFP^PER^* (R), *RFP^PER^ GFP^MIT^* (*RG*), and *RGB* after incubation on solid AMM for 48 h. (c) Biomass production (dry weight) of *RGB* and wt. Liquid cultures were incubated for 20 h at pH 5 and pH 7. The data illustrate the means for biological triplicates. Error bars indicate the standard deviations. *P* values were calculated by Student’s *t* test (two-tailed, unpaired): 0.38 and 0.32 for pH 5 and pH 7, respectively (reference, wt).

In the first step, we integrated an expression cassette encoding mKate2 carrying a C-terminal peroxisomal targeting sequence (PTS1, tripeptide SKL) (22) in the *fcyB* locus, yielding strain *RFP^PER^* (Δ*fcyB*::*mKate2^PER^*). In this strain, a second expression cassette encoding sGFP containing an N-terminal mitochondrial targeting sequence derived from citrate synthase (23) was targeted into the *fcyA* locus, yielding *RFP^PER^ GFP^MIT^* strain (Δ*fcyB*::*mKate2^PER^* Δ*fcyA*::*sGFP^MIT^*). In the last step, an expression cassette encoding mTagBFP2 with expected cytoplasmic localization was integrated into the *uprt* locus yielding strain *RFP^PER^ GFP^MIT^ BFP^CYT^* (Δ*fcyB*::*mKate2^PER^* Δ*fcyA*::*sGFP^MIT^* Δ*uprt*::*mTagBFP2^CYT^*). Multicolor laser scanning confocal microscopy confirmed expression of all three fluorescent proteins in *RFP^PER^ GFP^MIT^ BFP^CYT^* and localization to distinct subcellular compartments (Fig. 3 and Fig. S4). A noteworthy finding was that the simultaneous lack of FcyB, FcyA, and Uprt (*RFP^PER^ GFP^MIT^ BFP^CYT^* strain) affected neither growth (Fig. 3), virulence in a murine model of invasive aspergillosis, nor adaptation to various stress environments (Fig. S5).

10.1128/mBio.00230-20.4 FIG S4 Multicolor imaging of *RFP^PER^ GFP^MIT^ BFP^CYT^*. 3D reconstruction of single and merged channels for *mKate2^PER^* (peroxisomal), *sGFP^MIT^* (mitochondrial), and *mTagBFP2^CYT^* (cytosolic) is shown. Download FIG S4, DOCX file, 1.2 MB. Copyright © 2020 Birštonas et al. 2020 Birštonas et al. This content is distributed under the terms of the Creative Commons Attribution 4.0 International license.

10.1128/mBio.00230-20.5 FIG S5 Simultaneous lack of the three pyrimidine salvage pathway genes *fcyB*, *fcyA*, and *uprt* does not affect A. fumigatus virulence in a pulmonary murine model of aspergillosis as well as its capacity to adapt to different stress environments. (a) Survival of female outbred CD-1 mice immunosuppressed with cortisone acetate and intranasally infected with 2 × 10^5^ conidia in 20 μl PBS is shown as Kaplan-Meier curves. Δ*fcyB* Δ*fcyA* Δ*uprt.1* and Δ*fcyB* Δ*fcyA* Δ*uprt.2* represent two independent knock-in transformants lacking FcyB, FcyA, and Uprt. Analysis by log rank test showed no significant differences (*P* > 0.05 in comparison to wt and comparison between mutants; mock infected animals [5 mice per group], infected animals [10 mice per group]). (b) Phenotypic analysis of *RFP^PER^ GFP^MIT^ BFP^CYT^* (*RGB*) during various stress conditions, including oxidative stress (H_2_O_2_), high copper (0.5 mM CuSO_4_), high cobalt (0.5 mM CoSO_4_), iron deprivation (-Fe), zinc deprivation (-Zn), osmotic stress (1 M sorbitol), high temperature (48°C), as well as antifungal-drug-associated stress (0.1 μg/ml caspofungin [CASPO]). Therefore, strains were grown on solid AMM for 48 h at 37°C (left). To monitor voriconazole (VORI) and amphotericin B (AMPHO) resistance, MIC values were determined following EUCAST guidelines (right) (Subcommittee on Antifungal Susceptibility Testing of the ESCMID European Committee for Antimicrobial Susceptibility Testing, Clin Microbiol Infect 14:982–984, 2008, https://doi.org/10.1111/j.1469-0691.2008.02086.x). Download FIG S5, DOCX file, 0.6 MB. Copyright © 2020 Birštonas et al. 2020 Birštonas et al. This content is distributed under the terms of the Creative Commons Attribution 4.0 International license.

Collectively, these data demonstrate the feasibility of sequential use of endogenously encoded counterselectable markers *fcyB*, *fcyA*, and *uprt* for integration of up to three DOIs without adverse effects on A. fumigatus growth and virulence.

### Endogenous counterselectable markers can be used for the integration of biotechnologically relevant, large DNA fragments.

**FIG 4:**
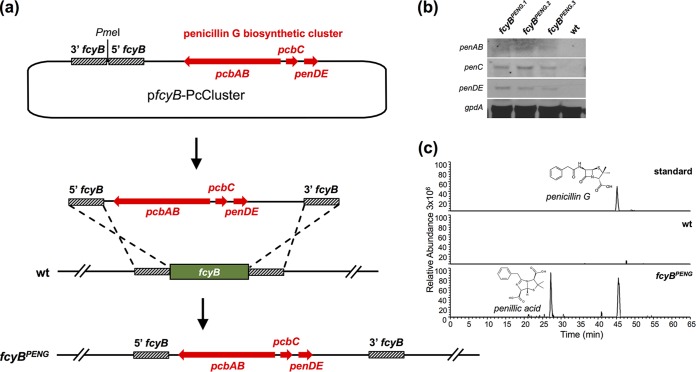
Genomic insertion of the PcCluster-transformed A. fumigatus into a penicillin G producer. (a) To facilitate genomic integration of the PcCluster at the *fcyB* locus, the plasmid p*fcyB*-PcCluster comprising the respective DOI (17 kb) as well as *fcyB* 5′ and 3′ NTRs was generated. Linearization of this plasmid with PmeI allows homologous recombination-based replacement of *fcyB* coding sequence with DNA containing the PcCluster. (b) Expression of functional *pcbAB*, *pcbC* and *penDE* was monitored in three independent transformants using Northern blot analysis (*gpdA* was used as reference). (c) LC-MS/MS-extracted ion chromatograms of penicillin G (peak at 45 min) and its degradation product penillic acid (peak at 27 min) in the culture supernatant of *fcyB^PENG^* strain after shaking incubation for 48 h at 25°C. wt served as a negative control.

Fungi play important roles as cell factories in food industry as well as medicine. Pursuing a potential biotechnological approach using the described selection method, we tested whether we can integrate the 17-kb penicillin biosynthetic cluster (PcCluster) of P. chrysogenum into the genome of A. fumigatus. Therefore, a knock-in plasmid was constructed comprising the PcCluster as well as 5′ and 3′ *fcyB* NTRs. Linearization of the plasmid with PmeI allowed double crossover homologous recombination-mediated replacement of *fcyB* by the PcCluster, as illustrated in Fig. 4a and Fig. S6a.

10.1128/mBio.00230-20.6 FIG S6 Generation of pfcyB-PcCluster and fragmentation patterns of penicillin G and penillic acid. (a) After amplification of fcyB 5′- (5′ fcyB-FW/-RV) and 3′-NTRs (3′ fcyB-FW/-RV) from A. fumigatus genomic DNA (Af-gDNA) (i), the purified fragments were assembled (NEBuilder) into pUC19L (ii). The primers 5′ fcyB-FW and 3′ fcyB-RV contained an add-on sequence, including the PmeI restriction site. The resulting plasmid pfcyB was linearized by PCR amplification (iii) using primers BB-pfcyB-FW/RV. Two overlapping fragments comprising the penicillin G biosynthetic cluster were amplified from P. chrysogenum genomic DNA (Pc-gDNA) employing primer pairs PcFrag1-FW/RV and PcFrag2-FW/RV (iv). PcFrag1, PcFrag2, and linear pfcyB were assembled (v), giving rise to pfcyB-PcCluster. (b) Structure-specific fragmentation patterns of penicillin G (*m*/*z* 335.1060 → 160.04, 176.07, and 114.04; upper panel) and penillic acid (*m*/*z* 335.1060 → 289.10 and 128.05; lower panel) (F. Aldeek, D. Canzani, M. Standland, M. R. Crosswhite, W. Hammack, G. Gerard, J. M. Cook, J Agr Food Chem 64: 6100–6107, 2016, https://doi.org/10.1021/acs.jafc.5b06150) are illustrated. Download FIG S6, DOCX file, 0.4 MB. Copyright © 2020 Birštonas et al. 2020 Birštonas et al. This content is distributed under the terms of the Creative Commons Attribution 4.0 International license.

Subsequent to transformation of this construct in the wt (selection, 10 μg/ml 5FC, pH 5), we validated its site-specific integration at the *fcyB* locus (strain *fcyB^PENG^*; Fig. S1). Northern blot analysis confirmed expression of penicillin biosynthetic genes *pcbAB*, *pcbC*, and *penDE* in three transformants (Fig. 4b). Concomitant to successful expression of this heterologous gene cluster in A. fumigatus, we detected penicillin G and its degradation product penillic acid in culture supernatants using nano-scale liquid chromatography (nanoLC) mass spectrometry (Fig. 4c). Both substances have the same molecular weight of 335.1060 g/mol but can be differentiated due to structure-specific fragmentation during tandem mass spectrometry (MS/MS) (Fig. S6b).

Taken together, these results demonstrate that pyrimidine salvage-based selectable markers can be applied for the integration of large-size DNA fragments, including whole gene clusters.

### Pyrimidine salvage-based selectable markers can be utilized in Penicillium chrysogenum and Fusarium oxysporum.

To identify encoded CD and UPRT activities in other fungal species, we searched for A. fumigatus FcyA and Uprt orthologs in biotechnologically (Aspergillus niger, Aspergillus oryzae, P. chrysogenum, Komagataella phaffii [previously Pichia pastoris], S. cerevisiae, and Trichoderma reesei) and pathologically relevant fungal species (Candida albicans, Cryptococcus neoformans, and F. oxysporum). *In silico* inspection of the annotated genomes of these species revealed that 8 out of the 10 species analyzed harbor a putative ortholog of A. fumigatus FcyA and that all species possess a putative ortholog of A. fumigatus Uprt with overall sequence identities of ≥40% (see Table S1A in the supplemental material). Notably, all species encoding an FcyA ortholog were also found to encode an FcyB ortholog. The genetic coupling of these two features might indicate that their main function is the utilization of extracellular pyrimidines.

10.1128/mBio.00230-20.8 TABLE S1 Homologous proteins to A. fumigatus (A1163) FcyB, FcyA, and Uprt in fungal species with relevance in biotechnology, agriculture, or medicine identified by BLASTP analysis (https://blast.ncbi.nlm.nih.gov/Blast.cgi). (A) The best hits, showing ≥40% identity, are illustrated. (B) A combination of BLAST-based *in silico* analysis and susceptibility testing further suggested the presence or absence of A. fumigatus FcyB, FcyA, or Uprt orthologs. MICs of 10 fungal species were determined following EUCAST guidelines (Subcommittee on Antifungal Susceptibility Testing of the ESCMID European Committee for Antimicrobial Susceptibility Testing, Clin Microbiol Infect 14:982–984, 2008, https://doi.org/10.1111/j.1469-0691.2008.02086.x). All strains were uniformly incubated in RPMI at 30°C for 48 h, followed by visual assessment of MICs. Download Table S1, DOCX file, 0.03 MB. Copyright © 2020 Birštonas et al. 2020 Birštonas et al. This content is distributed under the terms of the Creative Commons Attribution 4.0 International license.

To confirm CD and UPRT activities in the species analyzed, we monitored 5FC and 5FU susceptibility profiles following a broth microdilution-based method according to EUCAST (24). In agreement with our homology search, species with predicted FcyA orthologs (CD activity) were susceptible to 5FC, while those without these orthologs were resistant to the drug (Table S1B). All strains were susceptible to 5FU, which is in accordance with *in silico*-predicted Uprt orthologs (UPRT activity).

**FIG 5:**
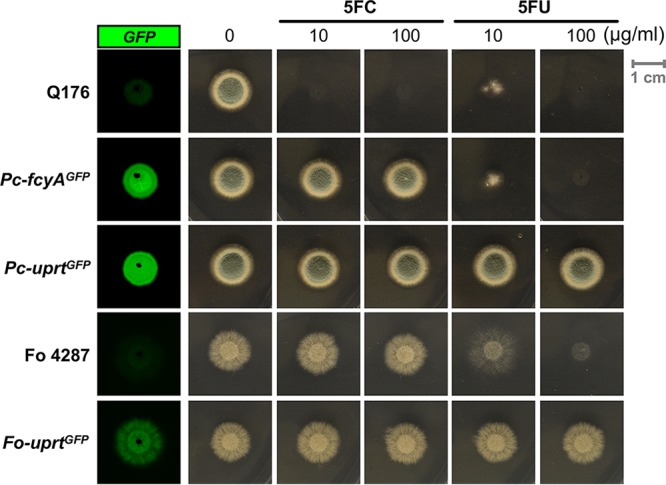
P. chrysogenum and F. oxysporum strains with replaced loci encoding components of the pyrimidine salvage pathway. GFP expression was visualized in the corresponding knock-in mutants in the absence of drugs. 5FC/5FU resistance phenotypes of GFP reporter strains lacking CD (*Pc-fcyA^GFP^*) or UPRT (*Pc-uprt^GFP^*, *Fo-uprt^GFP^*) activities. For both experiments, strains were grown on solid AMM (P. chrysogenum) and PDA (F. oxysporum) followed by 72 h of incubation at 25°C.

In the next step, we tested the applicability of the described selection strategy in P. chrysogenum and F. oxysporum. In agreement with the genomic data and 5FC/5FU susceptibility, P. chrysogenum expresses both CD (P. chrysogenum FcyA [Pc-FcyA], EN45_039280) and UPRT (Pc-Uprt, EN45_060980), while F. oxysporum lacks CD but expresses UPRT (F. oxysporum Uprt [Fo-Uprt], FOXG_01418). Employing the same protocol as used for A. fumigatus enabled the integration of GFP expression cassettes flanked by the 5′ and 3′ NTRs of the respective P. chrysogenum genes in both the *Pc-fcyA* and the *Pc-uprt* loci. In F. oxysporum, the same strategy enabled the targeting of a GFP expression cassette at the *Fo-uprt* locus. The presence and functionality of the GFP reporters were visualized as described above (Fig. 5). As observed for A. fumigatus knock-in mutants, the resistance profiles of P. chrysogenum and F. oxysporum knock-in strains were in accordance with the absence of individual salvage activities.

Taken together, these data indicate high evolutionary conservation of the described pyrimidine salvage enzymes within the fungal clade and demonstrate the suitability of the loci encoding these enzymes as markers for transformation selection in P. chrysogenum and F. oxysporum.

## DISCUSSION

In this study, we report the characterization and exploitation of multiple, endogenously encoded selectable markers for targeted genetic engineering. The marker genes encode activities mediating 5FC uptake (*fcyB*) and metabolization (*fcyA* and *uprt*) of 5FC or 5FU into cell-toxic nucleotides (Fig. 1a) (12). Genomic replacement of these genes by DOIs results in loss of the corresponding activities and can therefore be selected via 5FC or 5FU resistance. We validated the applicability of orthologous marker genes encoding pyrimidine salvage activities for their use as selectable markers in three fungal species (A. fumigatus, P. chrysogenum, and F. oxysporum) by targeted insertion of various fluorescent or enzymatic reporter genes (Fig. 2, 3, and 5). Mimicking a biotechnological application, we further introduced the 17-kb penicillin biosynthetic gene cluster from P. chrysogenum into A. fumigatus, which naturally is not capable of producing this secondary metabolite. In addition to their single use, we demonstrate the possibility of consecutive use of these markers in a single strain by generating an A. fumigatus mutant expressing three fluorescent proteins (mKate2, sGFP, and mTagBFP2) targeted to different subcellular compartments (Fig. 3a; see also Fig. S4 in the supplemental material). Importantly, these endogenous genes can be used in addition to traditional selectable markers, and the absence of all three genes (Δ*fcyB* Δ*fcyA* Δ*uprt*) affected neither growth nor virulence (Fig. 3b and Fig. S5) in A. fumigatus, which represents a prerequisite for downstream use of engineered strains. Similarly to *fcyB*, *fcyA*, and *uprt*, the orotidine-5′-decarboxylase-encoding gene *pyrG* illustrates a counterselectable marker, since loss of the corresponding gene function confers resistance to 5′-fluoroorotic acid. However, PyrG is essential for the *de novo* biosynthesis of pyrimidines, and its loss renders cells uracil auxotrophic (25, 26), which represents a major drawback in comparison to the markers described here.

Inspection of genomic sequences combined with 5FC/5FU susceptibility profiling of different fungal species playing important roles in biotechnology, medicine, or agriculture revealed the presence of *uprt* orthologs in all 10 species analyzed and *fcyB* as well as *fcyA* orthologs in 8 out of 10 species (see Table S1 in the supplemental material), indicating the broad applicability of the proposed selection method. Most likely, the number of endogenous counterselectable markers for DOI integration allows expansion beyond the genes characterized here. For instance, genes coding for components involved in 5FU uptake, such as orthologs of A. nidulans *furD* or S. cerevisiae *FUR4* (27, 28), represent potential candidates.

Employing pyrimidine salvage pathway-based endogenous markers, virtually any DOI can be inserted into a specific site in the target genome without apparent effects on growth phenotypes, demonstrating their huge potential for diverse applications in basic as well as applied research. The sequential use of marker genes enables the establishment of modular toolboxes, e.g., allowing site-directed insertion of multiple reporter genes for colocalization studies. Furthermore, the possibility of equipping strains with multiple DNA building blocks makes this technology particularly attractive for synthetic biology. Notably, the fact that marker genes have to be inactivated to allow selection based on 5FC/5FU resistance enforces targeted integration of DOIs. As the strategy described here avoids the use of foreign selection markers, the described genetic toolset further enables so-called “self-cloning.” Strains engineered this way are not considered genetically modified organisms (GMO) in some countries (29, 30). For this, only endogenously encoded DOIs can be used; examples include the increase of gene dosage or engineering of sequences such as promoter swapping. A further advantage of endogenous markers is the dispensability of antibiotic resistance genes, the use of which is generally discouraged in the manufacturing of medical or food-related products (31, 32), as it may promote horizontal transfer of resistance genes.

In summary, we characterized a powerful genetic toolbox enabling multiple, targeted genomic insertions of DOIs. Evolutionary conservation of the pyrimidine salvage pathway suggests broad applicability of the described marker genes. Thus, we anticipate that this technology will significantly advance genetic and metabolic engineering of diverse organisms.

## MATERIALS AND METHODS

### Growth conditions and fungal transformation.

Plate growth assays analyzing A. fumigatus and P. chrysogenum were conducted using solid *Aspergillus* minimal medium (AMM) (ammonium tartrate was used as nitrogen source, glucose as carbon source) (33), and for F. oxysporum, solid PDA was employed. Therefore, 10^4^ spores of each strain were point inoculated on agar plates. Low-pH medium contained 100 mM citrate buffer (pH 5), and neutral-pH medium contained 100 mM morpholinepropanesulfonic acid (MOPS) buffer (pH 7). If not stated in the text or images, plate growth assays were performed at pH 5. For strains carrying *xylP* promoter (*PxylP*)-driven reporter genes (*sGFP*, *mKate2^PER^*, *sGFP^MIT^*, *lacZ*), the medium was supplemented with 0.5% xylose to induce gene expression. Environmental and drug-related stress was assayed by either adding high concentrations of H_2_O_2_, copper, cobalt, or sorbitol, omitting iron or zinc micronutrient, incubating at 48°C, or supplementing medium with caspofungin, voriconazole, or amphotericin B (see Fig. S5 in the supplemental material).

For fungal manipulations, 2 μg DNA of each construct was transformed into protoplasts of the respective recipient. For the regeneration of transformants, solid AMM (A. fumigatus and P. chrysogenum) or PDA (F. oxysporum) supplemented with 342 g/liter or 200 g/liter sucrose, respectively, were used. Selection procedures using conventional selectable marker genes (*hph*, *ble*) were conducted as described previously for A. fumigatus (16).

### Deletion of A. fumigatus *fcyA* and *uprt*.

The strains and primers used in this study are listed in Tables S2 and S3 in the supplemental material. Coding sequences of *fcyA* and *uprt* were disrupted in the wild type (wt) (A1160P+) using hygromycin B and zeocin resistance cassettes, respectively. Therefore, deletion constructs comprising approximately 1 kb of 5′ and 3′ nontranslated regions (NTRs) linked to the central antibiotic resistance cassette were generated using fusion PCR as previously described (21). Correct integration of constructs was confirmed by Southern blot analyses (Fig. S1).

10.1128/mBio.00230-20.9 TABLE S2 Strains used in this study. Download Table S2, DOCX file, 0.03 MB. Copyright © 2020 Birštonas et al. 2020 Birštonas et al. This content is distributed under the terms of the Creative Commons Attribution 4.0 International license.

10.1128/mBio.00230-20.10 TABLE S3 Oligonucleotides used in this study. Download Table S3, DOCX file, 0.01 MB. Copyright © 2020 Birštonas et al. 2020 Birštonas et al. This content is distributed under the terms of the Creative Commons Attribution 4.0 International license.

### Generation of A. fumigatus knock-in strains.

Knock-in constructs for A. fumigatus *fcyB*, *fcyA*, and *uprt* loci, P. chrysogenum loci *Pc-fcyA* and *Pc-uprt*, as well as F. oxysporum *Fo-uprt* were generated similarly to the gene deletion fragments described above using fusion PCR. Here, instead of the antibiotic resistance cassettes, DNAs of interest (DOIs) (for reporter templates, see also Fig. S7) were connected to approximately 1-kb 5′ and 3′ NTRs of the respective locus (Fig. 2a).

10.1128/mBio.00230-20.7 FIG S7 Plasmid templates used for the generation of the different DOIs transformed in this work. For the amplification of the reporter cassettes comprising *sGFP*, *lacZ*, *mKate2^PER^*, and *sGFP^MIT^* from the “pX” plasmids pX-sGFP, pX-mKate2^PER^, pX-sGFP^MIT^, and pX-lacZ, the primer pair P1/P2 was used. An mTagBFP2-containing cassette was amplified from pAN-mTagBFP2 using primers hph-FW/hph-RV. For F. oxysporum, the GFP reporter cassette was amplified from pgpdA-GFP using primers FoGFP-Fw/Rv. In “pX” plasmids, the reporter genes are under control of the xylose-inducible promoter *PxylP*; in the other two plasmids, the reporter genes are driven by the constitutive *gpdA* promoter derived from A. nidulans (P. J. Punt, M. A. Dingemanse, B. J. Jacobs-Meijsing, P. H. Pouwels, C. A. van den Hondel, Gene 69:49–57, 1988, https://doi.org/10.1016/0378-1119(88)90377-0). Other abbreviations: MTS, mitochondrial targeting sequence; PTS, peroxisomal targeting sequence. Download FIG S7, DOCX file, 0.4 MB. Copyright © 2020 Birštonas et al. 2020 Birštonas et al. This content is distributed under the terms of the Creative Commons Attribution 4.0 International license.

### LacZ-based colorimetric assay and fluorescence imaging.

For the detection of LacZ activity (conversion of 5-bromo-4-chloro-3-indolyl-β-d-galactopyranoside [X-Gal] into the blue compound 5,5′-dibromo-4,4′-dichlor-indigo) (34), a 5-ml layer of a 1 mM X-Gal−1% agar−1% *N*-lauroylsarcosin solution was poured over fungal colonies. GFP expression of fungal colonies was visualized using the laser scanner Typhoon FLA9500 (excitation [Ex], 473 nm; emission [Em], ≥510 nm).

Images of *RFP^PER^ GFP^MIT^ BFP^CYT^* were taken using an HC PL APO CS2 63×/1.30 glycerol objective on an SP8 confocal microscope (Leica Microsystems, Wetzlar, Germany) equipped with a 80-MHz pulsed white light laser (WLL) and a 405-nm CW diode laser (405-nm diode) according to Nyquist sampling. Gating of the red signal only was used in order to remove unspecific red autofluorescence. Images of mKate2^PER^ (Ex, 588-nm WLL; Em, 598 to 750 nm; gating, 0.2. to 8 ns), sGFP^MIT^ (Ex, 489-nm WLL; Em, 499 to 578 nm), and mTagBFP^CYT^ (Ex, 405-nm diode; Em, 415 to 479 nm) were processed using ImageJ (Fig. 3a). For the generation of three-dimensional (3D) reconstructions (Fig. S4), z-stacks, acquired under the same imaging conditions using a z-interval of 180 nm, were deconvolved using the CMLE algorithm of Huygens Professional version 18.10 (Scientific Volume Imaging, Amsterdam, The Netherlands) and further processed with Imaris 9.3.0 (Bitplane, Zurich, Switzerland).

### Expression analysis of penicillin biosynthetic genes and detection of penicillin G in culture supernatants.

Northern blot analysis was conducted as described previously, using digoxigenin-labeled probes (35).

To detect the potential production of penicillin G, strains were grown in AMM for 48 h at 25°C, and 2 ml of culture supernatant was extracted with 1 volume of butyl acetate. The organic phase was collected in a new reaction tube and dried using a centrifugal vacuum concentrator (speed-vac). Nano-scale liquid chromatography-mass spectrometry (nanoLC-MS)-based detection of penicillin G was conducted using an UltiMate 3000 nano-scale high-performance liquid chromatography (HPLC) system coupled to a Q Exactive HF mass spectrometer (Thermo Scientific, Bremen, Germany). The samples were separated on a homemade fritless fused-silica microcapillary column (100-μm inner diameter [i.d.] by 280-μm outer diameter [o.d.] by 19-cm length) packed with 2,4-μm reversed-phase C18 material (Reprosil). Solvents for HPLC were 0.1% formic acid (solvent A) and 0.1% formic acid in 85% acetonitrile (solvent B). The gradient profile was as follows: 0 to 4 min, 4% solvent B; 4 to 57 min, 4 to 35% solvent B; 57 to 62 min, 35 to 100% solvent B; and 62 to 67 min, 100% solvent B. The flow rate was 300 nl/min.

Mass spectra were acquired in positive ion mode applying a precursor scan over the *m/z* range 50 to 500 in the FT analyzer. The ions at *m/z* = 335.1060 were selected from this precursor scan for tandem MS (MS/MS) fragmentation in the linear ion trap.

### Murine infection model.

Specific-pathogen-free female outbred CD-1 mice (18 to 20 g; 6 to 8 weeks old; Charles River, Germany) were housed under standard conditions in individually ventilated cages and supplied with normal mouse chow and water *ad libitum*. All animals were cared for in accordance with the European animal welfare regulations, and experiments were approved by the responsible federal/state authority and ethics committee in accordance with the German animal welfare act (permit no. 03-027/16). Mice were immunosuppressed with cortisone acetate and intranasally infected with 2 × 10^5^ conidia in 20 μl phosphate-buffered saline (PBS) as described before (36). Infected animals were monitored twice daily and humanely sacrificed if moribund (defined by a score, including weight loss, piloerection, behavior, and respiratory symptoms).
