# Supplementary material for: Multiplex Genetic Engineering Exploiting Pyrimidine Salvage Pathway-Based Endogenous Counterselectable Markers
Source: mBio. 2020 Apr 7;11(2):e00230-20. doi: 10.1128/mBio.00230-20 (PMC7157766; doi:10.1128/mBio.00230-20)
Supplement: FIG S1 [file mBio.00230-20-sf001.docx]

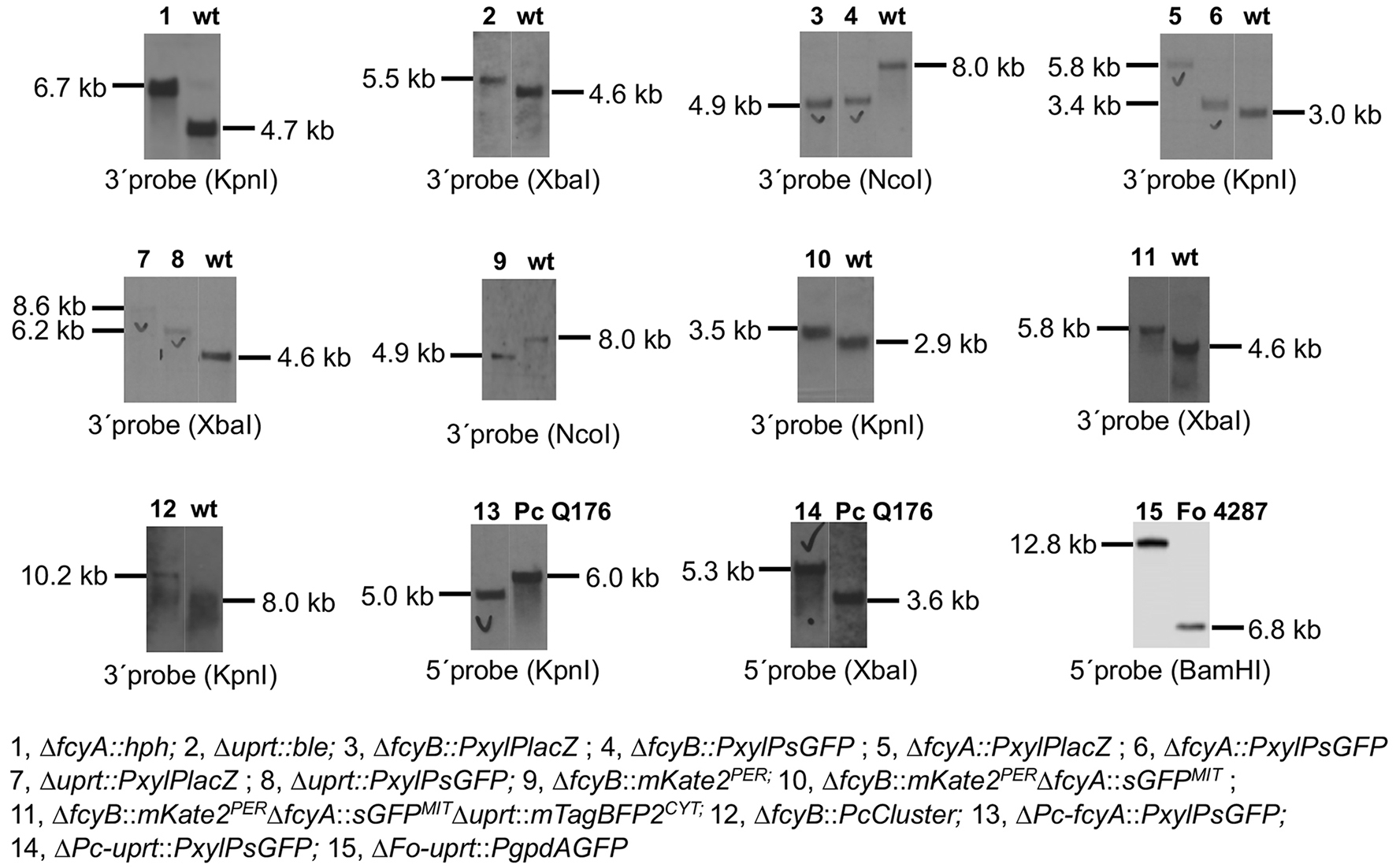


Fig. S1 **Southern blot analysis of strains generated in this work.** In each blot a representative transformant is compared to the respective recipient strain; wt, *A. fumigatus* A1160P+; Pc, *Penicillium chrysogenum*, Fo, *Fusarium oxysporum*.
