# Supplementary material for: Multiplex Genetic Engineering Exploiting Pyrimidine Salvage Pathway-Based Endogenous Counterselectable Markers
Source: mBio. 2020 Apr 7;11(2):e00230-20. doi: 10.1128/mBio.00230-20 (PMC7157766; doi:10.1128/mBio.00230-20)
Supplement: FIG S2 [file mBio.00230-20-sf002.docx]

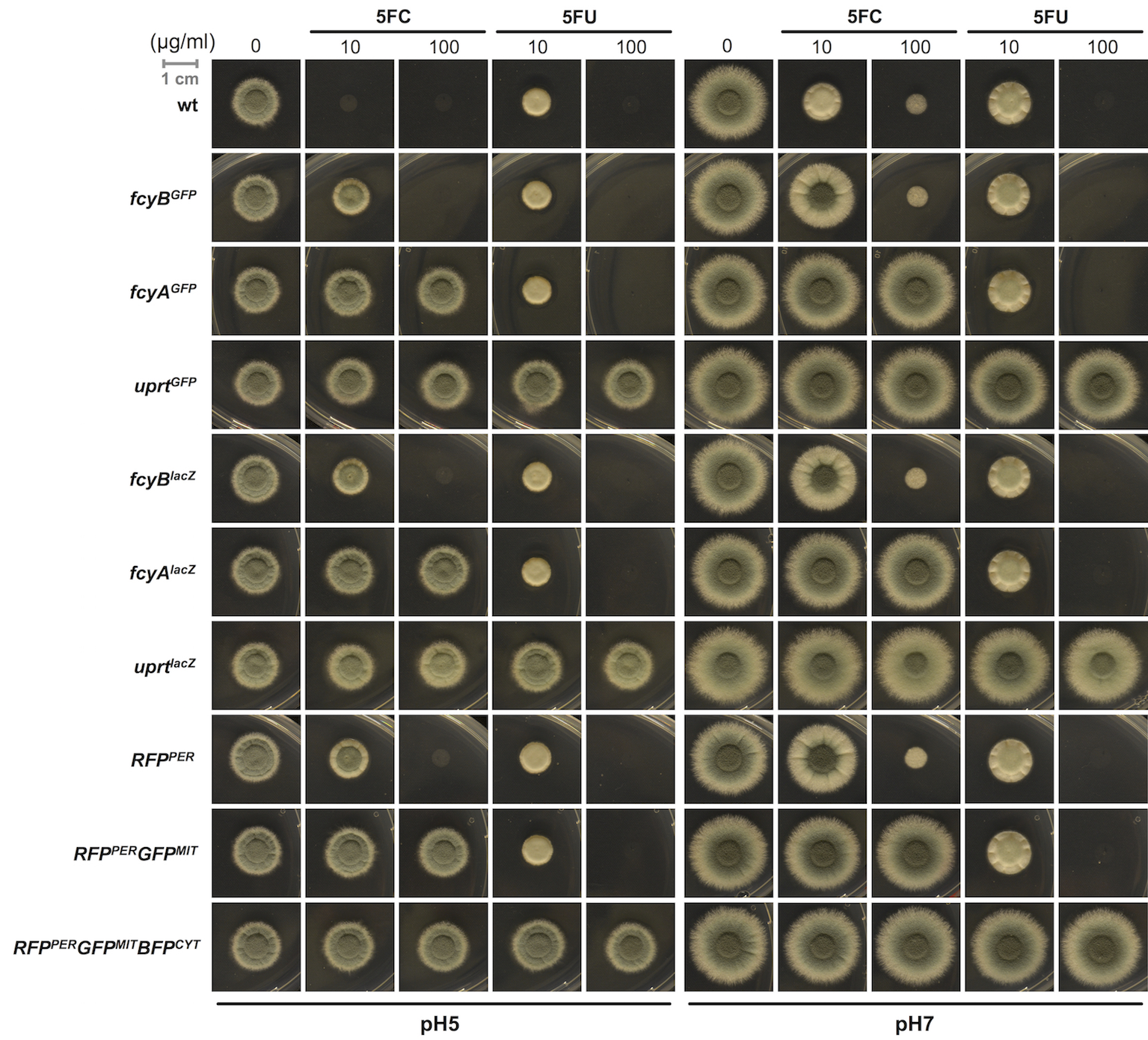
Fig. S2 **Plate growth based** **5FC/5FU susceptibility testing of *A. fumigatus* GFP and LacZ knock-in strains as well as *RFP^PER^GFP^MIT^BFP^CYT^* and its progenitor strains.** Strain were point inoculated on solid AMM at both pH5 and pH7 and incubated for 48 h at 37°C. Resistance phenotypes of all mutants analyzed were in accordance with the absence of individual salvage activities.
