# Supplementary material for: Multiplex Genetic Engineering Exploiting Pyrimidine Salvage Pathway-Based Endogenous Counterselectable Markers
Source: mBio. 2020 Apr 7;11(2):e00230-20. doi: 10.1128/mBio.00230-20 (PMC7157766; doi:10.1128/mBio.00230-20)
Supplement: FIG S3 [file mBio.00230-20-sf003.docx]

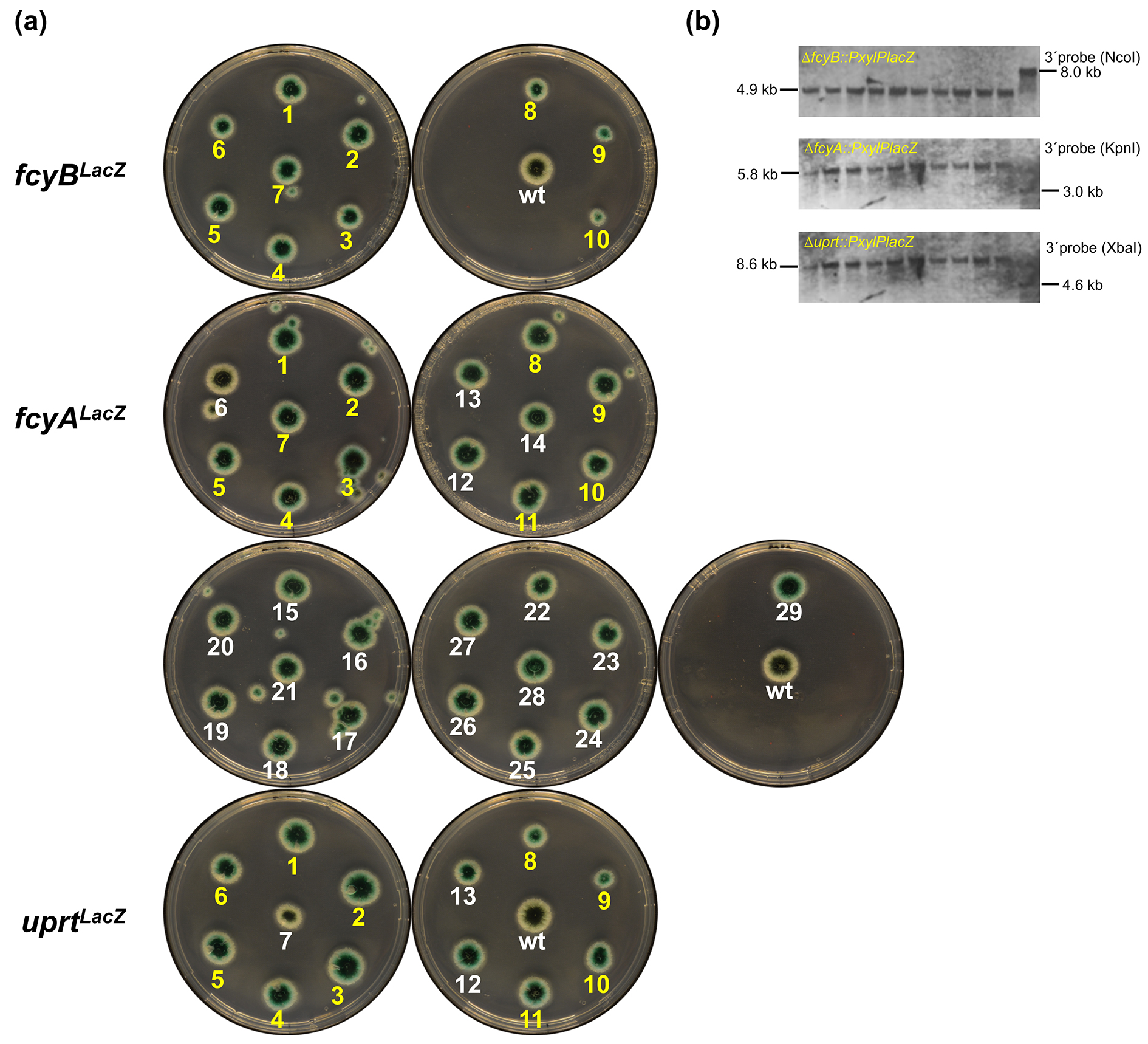


Fig. S3 **β-galactosidase staining to screen for LacZ-positive transformants.** After determining LacZ activities of each transformant (a), 10 transformants per locus showing LacZ-positive phenotypes (yellow numbers) were subject to Southern blot analysis (b). Strains were grown for 48 h at 37 °C on solid AMM before pouring an additional 5ml-layer of X-Gal containing agar on the top of colonies.
