# Supplementary material for: Multiplex Genetic Engineering Exploiting Pyrimidine Salvage Pathway-Based Endogenous Counterselectable Markers
Source: mBio. 2020 Apr 7;11(2):e00230-20. doi: 10.1128/mBio.00230-20 (PMC7157766; doi:10.1128/mBio.00230-20)
Supplement: FIG S4 [file mBio.00230-20-sf004.docx]

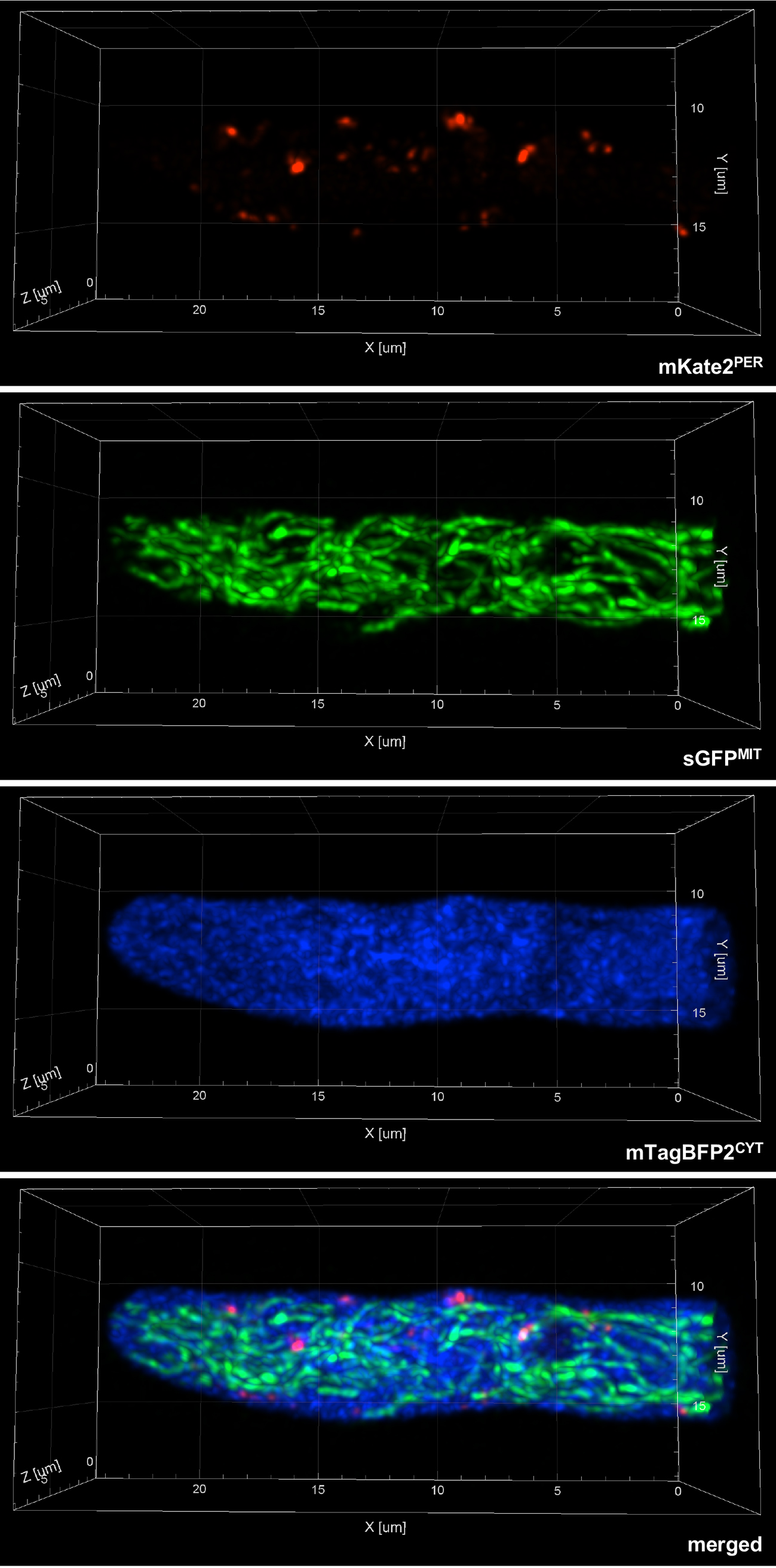


Fig. S4 **Multicolor imaging of *RFP^PER^GFP^MIT^BFP^CYT^*.** 3D reconstruction of single and merged channels for *mKate2^PER^* (peroxisomal), *sGFP^MIT^* (mitochondrial) and *mTagBFP2^CYT^* (cytosolic).
