# Supplementary material for: Multiplex Genetic Engineering Exploiting Pyrimidine Salvage Pathway-Based Endogenous Counterselectable Markers
Source: mBio. 2020 Apr 7;11(2):e00230-20. doi: 10.1128/mBio.00230-20 (PMC7157766; doi:10.1128/mBio.00230-20)
Supplement: FIG S5 [file mBio.00230-20-sf005.docx]

Fig. S5 **Simultaneous lack of the three pyrimidine salvage pathway genes *fcyB*, *fcyA* and *uprt* does not affect *A. fumigatus* virulence in a pulmonary murine model of aspergillosis as well as its capacity to adapt to different stress environments.** (a) Survival of female outbreed CD-1 mice immunosuppressed with cortisone acetate and intranasally infected with 2 x 105 conidia in 20 μl PBS is shown as Kaplan-Meier curves. ∆*fcyB*∆*fcyA*∆*uprt.1* and ∆*fcyB*∆*fcyA*∆*uprt.2* represent two independent knock-in transformants lacking FcyB, FcyA and Uprt. Analysis by log-rank test showed no significant differences (p > 0.05 in comparison to wt and comparison between mutants; mock infected n=5, infected animals n=10/group). (b) Phenotypic analysis of *RFP^PER^GFP^MIT^BFP^CYT^* (*RGB*) during various stress conditions including oxidative stress (H_2_O_2_), high copper (0.5 mM CuSO_4_), high cobalt (0.5 mM CoSO_4_), iron deprivation (-Fe), zinc deprivation (-Zn), osmotic stress (1M sorbitol), high temperature (48 °C) as well as antifungal drug associated stress (0.1 µg/ml caspofungin, CASPO). Therefore, strains were grown on solid AMM for 48 h at 37 °C (left). To monitor voriconazole (VORI) and amphotericin B (AMPHO) resistance, MIC values were determined following EUCAST guidelines (right) (Subcommittee on Antifungal Susceptibility Testing of the EECfAST 2008).

Subcommittee on Antifungal Susceptibility Testing of the EECfAST. 2008. Clinical microbiology and infection : the official publication of the European Society of Clinical Microbiology and Infectious Diseases 14: 982-984.
