# Supplementary material for: Multiplex Genetic Engineering Exploiting Pyrimidine Salvage Pathway-Based Endogenous Counterselectable Markers
Source: mBio. 2020 Apr 7;11(2):e00230-20. doi: 10.1128/mBio.00230-20 (PMC7157766; doi:10.1128/mBio.00230-20)
Supplement: FIG S6 [file mBio.00230-20-sf006.docx]

**
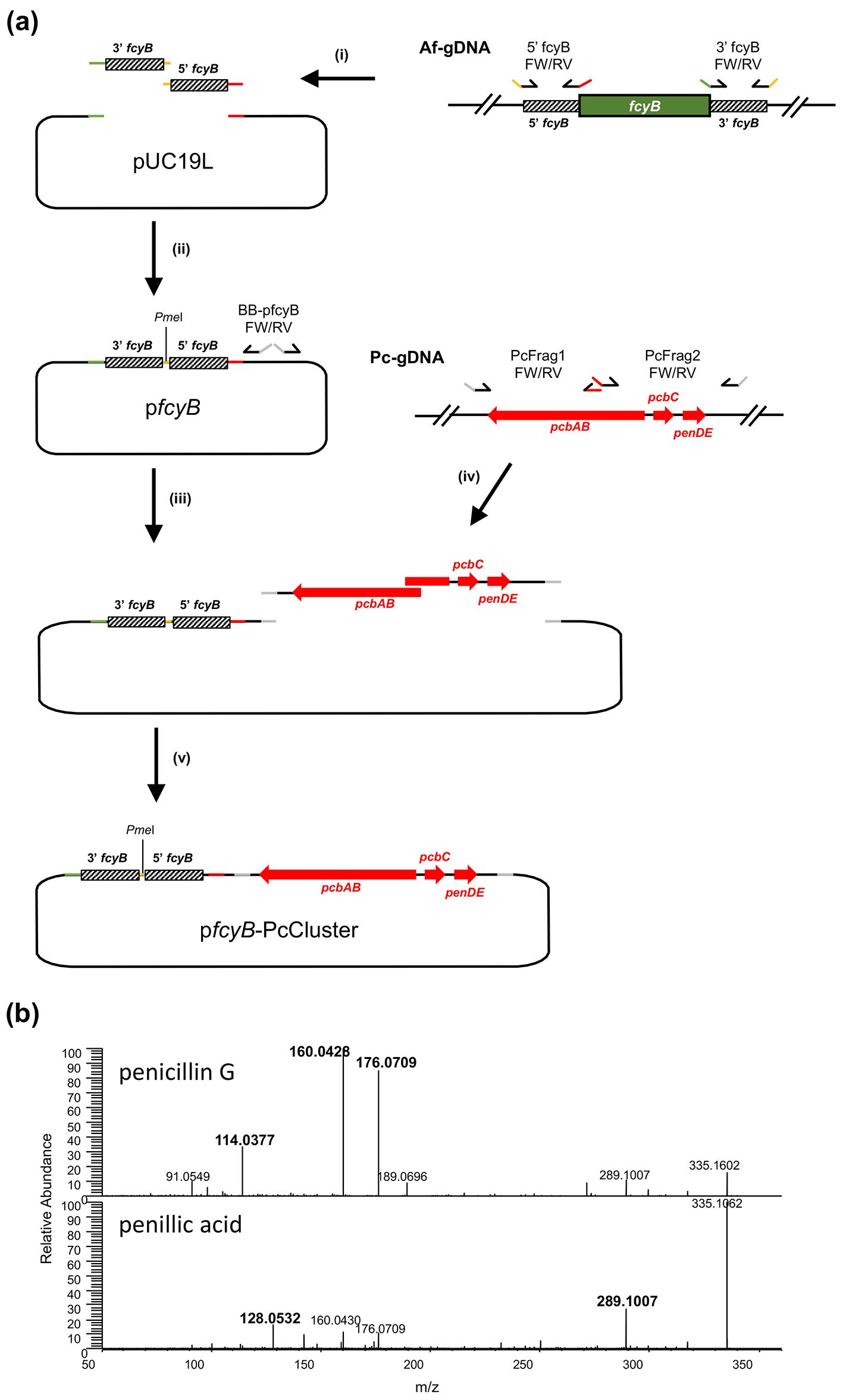
**

Fig. S6 **Generation of pfcyB-PcCluster and fragmentation patterns of penicillin G and penillic acid.** (a) After amplification of fcyB 5’- (5' fcyB-FW/-RV) and 3’-NTRs (3' fcyB-FW/-RV) from *A. fumigatus* genomic DNA (Af-gDNA) (i), the purified fragments were assembled (NEBuilder®) into pUC19L (ii). The primers 5' fcyB-FW and 3' fcyB-RV contained an add-on sequence including the *Pme*I restriction site. The yielding plasmid pfcyB was linearized by PCR amplification (iii) using primers BB-pfcyB-FW/RV. Two overlapping fragments comprising the penicillin G biosynthetic cluster were amplified from *P. chrysogenum* genomic DNA (Pc-gDNA) employing primer pairs PcFrag1-FW/RV and PcFrag2-FW/RV (iv). PcFrag1, PcFrag2 and linear pfcyB were assembled (v) giving rise to pfcyB-PcCluster. (b) Structure-specific fragmentation patterns of penicillin G (m/z 335.1060 -> 160.04, 176.07 and 114.04; upper panel) and penillic acid (m/z 335.1060 -> 289.10 and 128.05; lower panel) are illustrated (Aldeek et al. 2016).

Aldeek F, Canzani D, Standland M, Crosswhite MR, Hammack W, Gerard G, Cook JM. 2016. J Agr Food Chem 64: 6100-6107.
