# Supplementary material for: Multiplex Genetic Engineering Exploiting Pyrimidine Salvage Pathway-Based Endogenous Counterselectable Markers
Source: mBio. 2020 Apr 7;11(2):e00230-20. doi: 10.1128/mBio.00230-20 (PMC7157766; doi:10.1128/mBio.00230-20)
Supplement: TABLE S1 [file mBio.00230-20-st001.docx]

Table S1 **Homologous** **proteins to *A. fumigatus* (A1163) FcyB, FcyA and Uprt in fungal species with relevance in biotechnology, agriculture or medicine identified by BLASTP analysis (**[**https://blast.ncbi.nlm.nih.gov/Blast.cgi**](https://blast.ncbi.nlm.nih.gov/Blast.cgi)**).** (A) The best hits, showing > 40% identity, are illustrated. (B) A combination of BLAST based *in silico* analysis and susceptibility testing further suggested the presence or absence of *A. fumigatus* FcyB, FcyA or Uprt orthologs. MICs of 10 fungal species were determined following EUCAST guidelines (Subcommittee on Antifungal Susceptibility Testing of the EECfAST 2008). All strains were uniformly incubated in RPMI at 30 °C for 48 h followed by visual assessment of MICs.

| **FcyB** | **Species** | **Total score** | **Query coverage** | **E-value** | **Identity** | **Protein Accession** |
| --- | --- | --- | --- | --- | --- | --- |
|  | *Aspergillus fumigatus A1163* | 1060 | 100% | 0 | 100% | EDP54513.1 |
|  | *Aspergillus oryzae RIB40* | 855 | 100% | 0 | 80% | XP_001826247.1 |
|  | *Aspergillus niger ATCC 1015* | 818 | 100% | 0 | 77% | EHA22089.1 |
|  | *Penicillium chrysogenum* | 780 | 99% | 0 | 75% | KZN90676.1 |
|  | *Saccharomyces cerevisiae P283* | 415 | 98% | 2.00E-139 | 42% | EWH18811.1 |
|  | *Candida albicans SC5314* | 411 | 91% | 2.00E-138 | 46% | XP_714531.2 |
|  | *Saccharomyces cerevisiae P283* | 411 | 98% | 3.00E-138 | 43% | EWH18815.1 |
|  | *Komagataella phaffii GS115* | 395 | 99% | 3.00E-132 | 42% | XP_002493949.1 |
|  | *Cryptococcus neoformans var. grubii H99* | 320 | 98% | 5.00E-103 | 40% | XP_012052683.1 |
| **FcyA** | **Species** | **Total score** | **Query coverage** | **E-value** | **Identity** | **Protein Accession** |
|  | *Aspergillus fumigatus A1163* | 303 | 100% | 3.00E-107 | 100% | EDP55842.1 |
|  | *Aspergillus niger ATCC 1015* | 280 | 100% | 4.00E-98 | 91% | EHA26383.1 |
|  | *Penicillium chrysogenum* | 278 | 100% | 2.00E-97 | 91% | KZN93743.1 |
|  | *Aspergillus oryzae RIB40* | 275 | 95% | 4.00E-96 | 93% | XP_001819938.3 |
|  | *Komagataella phaffii GS115^#^* | 196 | 95% | 1.00E-64 | 63% | XP_002490927.1 |
|  | *Candida albicans SC5314* | 189 | 95% | 3.00E-62 | 61% | KHC73214.1 |
|  | *Saccharomyces cerevisiae P283* | 181 | 95% | 5.00E-59 | 61% | EWH15533.1 |
|  | *Cryptococcus neoformans var. grubii H99* | 140 | 93% | 2.00E-42 | 49% | XP_012046842.1 |
| **Uprt** | **Species** | **Total score** | **Query coverage** | **E value** | **Identity** | **Protein Accession** |
|  | *Aspergillus fumigatus A1163* | 496 | 100% | 1.00E-180 | 100% | EDP51298.1 |
|  | *Aspergillus niger ATCC 1015* | 464 | 99% | 4.00E-168 | 94% | EHA22482.1 |
|  | *Penicillium chrysogenum* | 450 | 99% | 3.00E-162 | 89% | KZN87537.1 |
|  | *Aspergillus oryzae RIB40* | 445 | 100% | 3.00E-160 | 90% | XP_023088768.1 |
|  | *Trichoderma reesei QM6a* | 397 | 98% | 4.00E-141 | 78% | XP_006967593.1 |
|  | *Fusarium oxysporum f. sp. lycopersici 4287* | 389 | 95% | 6.00E-139 | 78% | XP_018234120.1 |
|  | *Komagataella phaffii GS115^#^* | 301 | 87% | 7.00E-104 | 67% | XP_002489914.1 |
|  | *Cryptococcus neoformans var. grubii H99* | 301 | 85% | 1.00E-103 | 69% | XP_012050086.1 |
|  | *Candida albicans SC5314* | 298 | 90% | 2.00E-102 | 66% | XP_712023.1 |
|  | *Saccharomyces cerevisiae P283* | 294 | 87% | 4.00E-101 | 66% | EWH18153.1 |

**(A)**

*^#^ old species name Pichia pastoris* (Heistinger et al. 2018).

**(B)**

|  | **MIC (µg/ml)** | | | | **Activities** | | |
| --- | --- | --- | --- | --- | --- | --- | --- |
|  | **5FC** | | **5FU** | |  |  |  |
| **Species** | **pH5** | **pH7** | **pH5** | **pH7** | **FcyB** | **FcyA** | **Uprt** |
| *Aspergillus fumigatus* | 0.39 | 400 | 50 | 100 | ✔ | ✔ | ✔ |
| *Aspergillus niger* | 0.39 | 6.25 | 50 | 100 | ✔ | ✔ | ✔ |
| *Aspergillus oryzae* | 0.39 | 400 | 200 | 400 | ✔ | ✔ | ✔ |
| *Candida albicans* | 0.39 | 0.39 | 50 | 100 | ✔ | ✔ | ✔ |
| *Cryptococcus neoformans* | 0.39 | 25 | 6.25 | 6.25 | ✔ | ✔ | ✔ |
| ***Fusarium oxysporum*** | **>400** | **>400** | 400 | 400 |  |  | ✔ |
| *Komagataella phaffii* | 0.39 | 0.39 | 0.39 | 0.39 | ✔ | ✔ | ✔ |
| *Penicillium chrysogenum* | 0.39 | 3.12 | 3.12 | 3.12 | ✔ | ✔ | ✔ |
| *Saccharomyces cerevisiae* | 0.39 | 0.39 | 0.8 | 50 | ✔ | ✔ | ✔ |
| ***Trichoderma reesei*** | **>400** | **>400** | 100 | 100 |  |  | ✔ |

**Bold**, strains lacking susceptibility to 5FC under both tested conditions, suggesting the absence of CD activity. ✔, indicates the presence of the corresponding activity.

Heistinger L, Gasser B, Mattanovich D. 2018. Mol Cell Biol 38.

Subcommittee on Antifungal Susceptibility Testing of the EECfAST. 2008. Clinical microbiology and infection : the official publication of the European Society of Clinical Microbiology and Infectious Diseases 14: 982-984.
