# Supplementary material for: Multiplex Genetic Engineering Exploiting Pyrimidine Salvage Pathway-Based Endogenous Counterselectable Markers
Source: mBio. 2020 Apr 7;11(2):e00230-20. doi: 10.1128/mBio.00230-20 (PMC7157766; doi:10.1128/mBio.00230-20)
Supplement: TABLE S2 [file mBio.00230-20-st002.docx]

Table S2 **Strains used in this study.**

| Strain | **Genotype** | **Reference** |
| --- | --- | --- |
| ***A. fumigatus*** | | |
| **Characterization of 5FC activity determinants** | | |
| A1160P+ (wild-type)^#^ | ∆*ku80, pyrG+* | (Fraczek et al. 2013) |
| ∆*fcyB* | ∆*fcyB::hph* | (Gsaller et al. 2018) |
| ∆*fcyA* | ∆*fcyA::hph* | This study |
| ∆*uprt* | ∆*uprt::ble* | This study |
|  | | |
| **Proof-of-principle work in *A. fumigatus*** | | |
| *fcyB^GFP^* | ∆*fcyB::PxylPsGFP* | This study |
| *fcyB^lacZ^* | ∆*fcyB::PxylPlacZ* | This study |
| *fcyA^GFP^* | ∆*fcyA::PxylPsGFP* | This study |
| *fcyA^lacZ^* | ∆*fcyA::PxylPlacZ* | This study |
| *uprt^GFP^* | ∆*uprt::PxylPsGFP* | This study |
| *uprt^lacZ^* | ∆*uprt::PxylPlacZ* | This study |
|  | | |
| **Multicolor laser scanning microscopy** | | |
| *RFP^PER^* | ∆*fcyB*::*mKate2^PER^* | This study |
| *RFP^PER^GFP^MIT^* | ∆*fcyB*::*mKate2^PER^*∆*fcyA*::*sGFP^MIT^* | This study |
| *RFP^PER^GFP^MIT^BFP^CYT^* | ∆*fcyB*::*mKate2^PER^*∆*fcyA*::*sGFP^MIT^*∆*uprt*::*mTagBFP2^CYT^* | This study |
|  | | |
| **Introduction of the penicillin G biosynthetic cluster** | | |
| *fcyB^PENG^* | ∆*fcyB*::*PcCluster* | This study |
|  | | |
| **Implementation of the 5FC/5FU selection method in *P. chrysogenum* and *F. oxysporum*** | | |
| ***P. chrysogenum*** | | |
| Q176 (wild-type) |  | (Backus and Stauffer 1955) |
| *Pc-fcyA^GFP^* | ∆*Pc-fcyA::PxylPsGFP* | This study |
| *Pc-uprt^GFP^* | ∆*Pc-uprt::PxylPsGFP* | This study |
| ***F. oxysporum*** | | |
| Fol 4287 (wild-type) |  | (Ma et al. 2010) |
| *Fo-uprt^GFP^* | ∆*Fo-uprt::PgpdAGFP* | This study |
|  |  |  |
| **Strains used for the determination of MICs** | | |
| *A. niger CBS 120-49* | | (Bos et al. 1988) |
| *A. oryzae NS4* | | (Jin et al. 2004) |
| *C. albicans SC5314* | | (Maestrone and Semar 1968) |
| *C. neoformans H99* | | (Perfect et al. 1980) |
| *K. phaffii KM71* | | (Salamin et al. 2010) |
| *S. cerevisiae BY4742* | | (Brachmann et al. 1998) |
| *T. reesei QM9414* | | (Ghose and Sahai 1979) |

^#^ *A. fumigatus* knock-in strains have A1160P+ as genetic background.

Backus MP, Stauffer JF. 1955. Mycologia 47: 429-463.

Bos CJ, Debets AJ, Swart K, Huybers A, Kobus G, Slakhorst SM. 1988. Current genetics 14: 437-443.

Brachmann CB, Davies A, Cost GJ, Caputo E, Li J, Hieter P, Boeke JD. 1998. Yeast 14: 115-132.

Fraczek MG, Bromley M, Buied A, Moore CB, Rajendran R, Rautemaa R, Ramage G, Denning DW, Bowyer P. 2013. The Journal of antimicrobial chemotherapy 68: 1486-1496.

Ghose TK, Sahai V. 1979. Biotechnol Bioeng 21: 283-296.

Gsaller F, Furukawa T, Carr PD, Rash B, Jochl C, Bertuzzi M, Bignell EM, Bromley MJ. 2018. Antimicrob Agents Chemother 62.

Jin FH, Maruyama J, Juvvadi PR, Arioka M, Kitamoto K. 2004. FEMS microbiology letters 239: 79-85.

Ma LJ, van der Does HC, Borkovich KA, Coleman JJ, Daboussi MJ, Di Pietro A, Dufresne M, Freitag M, Grabherr M, Henrissat B et al. 2010. Nature 464: 367-373.

Maestrone G, Semar R. 1968. Naturwissenschaften 55: 87-88.

Perfect JR, Lang SD, Durack DT. 1980. Am J Pathol 101: 177-194.

Salamin K, Sriranganadane D, Lechenne B, Jousson O, Monod M. 2010. Appl Environ Microbiol 76: 4269-4276.
