# Supplementary material for: Multiplex Genetic Engineering Exploiting Pyrimidine Salvage Pathway-Based Endogenous Counterselectable Markers
Source: mBio. 2020 Apr 7;11(2):e00230-20. doi: 10.1128/mBio.00230-20 (PMC7157766; doi:10.1128/mBio.00230-20)
Supplement: TABLE S3 [file mBio.00230-20-st003.docx]

| **primer name** | **forward primer (5´→3´)** | **reverse primer (5´→3´)** | **PCR product** |
| --- | --- | --- | --- |
| ***(A) fcyA and uprt deletion and knock-in cassettes.*** | | | |
| fcyA-1/-2 | TTGAAACTCCGAGGAAGTCG | TAGTTCTGTTACCGAGCCGGTATGTGGATCCAGAGCGTCA | 5' fcyA |
| fcyA-3/-4 | GCTCTGAACGATATGCTCCCTTCGACAAAATGCCATTGAA | TACCTCCCCGAATACCATGA | 3' fcyA & 3' probe for Southern analysis |
| fcyA-N1/-N2 | CGAGTCGCCTTAAAATGAGC | GTGGATCGGTATGCAGGATT | fcyA::hph deletion; knock-in constructs |
| uprt-1/-2 | GGAAGGACAGGTACGCCATA | TAGTTCTGTTACCGAGCCGGCGGAGCACTCTGAAAATTGG | 5' uprt |
| uprt-3/-4 | GCTCTGAACGATATGCTCCCTCCCATCGTGTAGCGACATA | TACTACCTTCGCCCTCTGGA | 3' uprt & 3' probe for Southern analysis |
| uprt-N1/-N2 | TTTGAGCGATTAAGGTGCAA | GCCCCACTACTTGTTTCCAG | uprt::ble deletion; knock-in constructs |
| fcyB-3/-4 | GCTCTGAACGATATGCTCCCTGCGGTTTTTGGGTTTTATC | CACACTGGGTCTGAAGACGA | 3' probe for Southern analysis |
| ***(B) Amplification of reporter cassettes.*** | | | |
| P1/P2 | CCGGCTCGGTAACAGAACTACTGATGCGAGCAACAGTATGC | GGGAGCATATCGTTCAGAGCTGAGGGTTGAGTACGAGATTGG | reporter cassettes: sGFP*, lacZ, mKate2^PER^, sGFP^MIT^ |
| hph-FW/-RV | CCGGCTCGGTAACAGAACTAACGGCGTAACCAAAAGTCAC | GGGAGCATATCGTTCAGAGCTCTTGACGACCGTTGATCTG | hygR & zeoR cassette; mTagBFP2^CYT^ reporter cassette |
| FoGFP-FW/-RV | CGAGACCTAATACAGCCCCTA | CCTGTGCATTCTGGGTAAACG | GFP reporter cassette for *F. oxysporum* |
| * same cassette was used for the *P. chrysogenum* reporter | | | |
| ***(C) Generation of the PcCluster containing knock-in plasmid*** | | | |
| 5' fcyB-FW/-RV | TGTGGCGGCCGCGTTTAAACCGCTATCCCAGCAATAGAGC | TTACGCCAAGCTTGCATGCCACTGAGTCAATCCCCACCAC | 5' fcyB |
| 3' fcyB-FW/-RV | AGTGAATTCGAGCTCGGTACTGCGGTTTTTGGGTTTTATC | AGCGGTTTAAACGCGGCCGCCACACTGGGTCTGAAGACGA | 3' fcyB |
| BB-pfcyB-FW/-RV | TGTGAAATTGTTATCCGCTCACAA | AAACAGCTATGACCATGATTACGC | backbone pfcyB |
| PcFrag1-FW/-RV | AATCATGGTCATAGCTGTTTAAAGGGGAGAGAGCGAAAAG | GCATGGGGACAATCTCACTT | fragment 1 PcCluster |
| PcFrag2-FW/-RV | AAGTGAGATTGTCCCCATGCAG | GAGCGGATAACAATTTCACACGCGTGATATCCTGTCTTCA | fragment 2 PcCluster |
| ***(D) Generation of the knock-in constructs for P. chrysogenum and F. oxysporum*** | | | |
| Pc-fcyA-1/-2 | TGACCTTGATGGCATCTGAA | TAGTTCTGTTACCGAGCCGGTCAGTGCGGGCTACAGAGTA | 5' Pc-fcyA & 5' probe for Southern analysis |
| Pc-fcyA-3/-4 | GCTCTGAACGATATGCTCCCGGCCTGCACATATCATAGCC | AGCCGTAAAATTCGCATCAC | 3' Pc-fcyA |
| Pc-fcyA-N1/-N2 | GTCGAGGTGCTCAATGTGAA | TTGTTTTGACTTCCCCTTCG | Pc-fcyA knock-in construct |
| Pc-uprt-1/-2 | GGACAGTTTGGACAATGCAG | TAGTTCTGTTACCGAGCCGGTTTGAAGGGCAAGAGTCCAG | 5' Pc-uprt & 5' probe for Southern analysis |
| Pc-uprt-3/-4 | GCTCTGAACGATATGCTCCCACCACGTTGAAAGGAGCATC | AGACCGTGGAAGTTGGTCAG | 3' Pc-uprt |
| Pc-uprt-N1/-N2 | TTTTGCAAGGGTCGAGAAAG | CAGTTCTTGCCCTGGATCTC | Pc-uprt knock-in construct |
| Fo-uprt-1/-2 | CATACGTCACCACCTTGC | GTTGTAGGGGCTGTATTAGGTCTCGGCTGTTGTTAGTGTTCGAGG | 5' Fo-uprt & 5' probe for Southern analysis |
| Fo-uprt-3/-4 | GAGTCGTTTACCCAGAATGCACAGGGAAGGAATCAGCGCAAAG | CACGTATAGAATCACGGAGG | 3' Fo-uprt |
| Fo-uprt-N1/-N2 | GACGCCATAGTGTGCTC | GCTTGATGCATGCACTAG | Fo-uprt knock-in construct |

Table S3 **Oligonucleotides used in this study.**
