# Supplementary material for: Multiplex Genetic Engineering Exploiting Pyrimidine Salvage Pathway-Based Endogenous Counterselectable Markers
Source: mBio. 2020 Apr 7;11(2):e00230-20. doi: 10.1128/mBio.00230-20 (PMC7157766; doi:10.1128/mBio.00230-20)
Supplement: FIG S7 [file mBio.00230-20-sf007.docx]

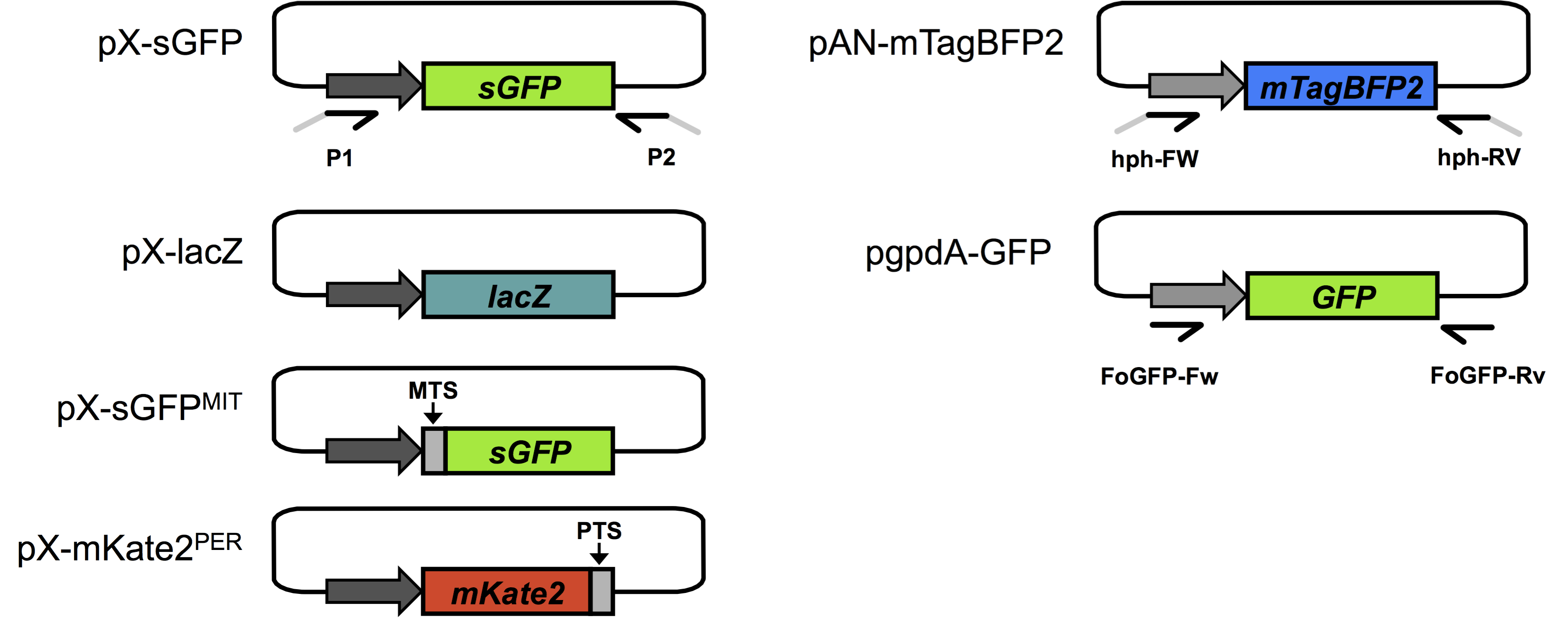


Fig. S7 **Plasmid templates used for the generation of the different DOIs transformed in this work.** For the amplification of the reporter cassettes comprising *sGFP*, *lacZ*, *mKate2^PER^*, *sGFP^MIT^* from the ‘pX’ plasmids pX-sGFP, pX-mKate2^PER^, pX-sGFP^MIT^, pX-lacZ the primer pair P1/P2 was used. An mTagBFP2 containing cassette was amplified from pAN-mTagBFP2 using primers hph-FW/hph-RV. For *F. oxysporum*, the GFP reporter cassette was amplified from pgpdA-GFP using primers FoGFP-Fw/Rv. In ‘pX’ plasmids, the reporter genes are under control of the xylose-inducible promoter *PxylP*; in the other two plasmids, the reporter genes are driven by the constitutive *gpdA* promoter derived from *A. nidulans* (Punt et al. 1988). Other abbreviations: MTS, mitochondrial targeting sequence; PTS, peroxisomal targeting sequence.

Punt PJ, Dingemanse MA, Jacobs-Meijsing BJ, Pouwels PH, van den Hondel CA. 1988. Gene 69: 49-57.
